# Bronchoalveolar lavage cell percentages as diagnostic markers of immune checkpoint inhibitor pneumonitis

**DOI:** 10.3389/fmed.2025.1582714

**Published:** 2025-06-02

**Authors:** Mahnoor Mir, Felipe Soto, Pedro Antonio Amezcua Gomez, Rodrigo Del Rio Arroyo, Adarsh Suresh, Amber Su, Qiong Gan, John Stewart, Roberto Adachi, Diwakar D. Balachandran, Lara Bashoura, Roberto F. Casal, Burton F. Dickey, George A. Eapen, Scott E. Evans, Horiana Grosu, Carlos A. Jimenez, Julie Lin, David E. Ost, Bruce F. Sabath, Vickie R. Shannon, Aung Naing, Jianjun Gao, Jia Wu, Karthik Suresh, Saadia A. Faiz, Mehmet Altan, Ajay Sheshadri

**Affiliations:** ^1^Division of Critical Care, Pulmonary, and Sleep Medicine, McGovern Medical School at UTHealth Houston, Houston, TX, United States; ^2^School of Medicine, Tecnológico de Monterrey, Monterrey, Mexico; ^3^Texas A&M School of Medicine, Houston, TX, United States; ^4^Department of Pulmonary Medicine, The University of Texas MD Anderson Cancer Center, Houston, TX, United States; ^5^Department of Pathology, The University of Texas MD Anderson Cancer Center, Houston, TX, United States; ^6^Department of Investigational Cancer Therapeutics, The University of Texas MD Anderson Cancer Center, Houston, TX, United States; ^7^Division of Cancer Medicine, Department of Genitourinary Medical Oncology, Houston, TX, United States; ^8^Department of Imaging Physics, The University of Texas MD Anderson Cancer Center, Houston, TX, United States; ^9^Department of Pulmonary and Critical Care Medicine, Johns Hopkins University, Baltimore, MD, United States; ^10^Department of Thoracic Oncology, The University of Texas MD Anderson Cancer Center, Houston, TX, United States

**Keywords:** immune check inhibitor (ICI), BAL (bronchoalveolar lavage), lymphocytosis, pneumonitis, eosinophilia, cell percentage

## Abstract

**Introduction:**

Diagnostic biomarkers for immune checkpoint inhibitor pneumonitis (ICIP) are lacking. Bronchoalveolar lavage (BAL) lymphocytosis has been associated with ICIP, but studies have not evaluated BAL lymphocytosis as a diagnostic biomarker for ICIP.

**Purpose:**

This study aimed to measure the association of BAL immune cell percentage with ICIP and test its performance as a diagnostic biomarker.

**Methods:**

We performed a retrospective chart review of 476 patients treated with ICIs for solid organ or hematologic malignancies who underwent BAL between 2016 and 2022. Two independent reviewers, blinded to the results of BAL cell percentage, confirmed the diagnosis of ICIP or other conditions (e.g., pneumonia) based on clinical history and radiology. We constructed logistic regression models to assess the relationship between BAL lymphocyte, eosinophil, and neutrophil percentages and the diagnosis of pneumonitis, and the area under the receiver-operator curves (AUROC) was used to assess their discriminatory function. We measured the association of BAL immune cell percentages with 1-year overall survival using Cox proportional hazard models adjusted for age and cancer diagnosis.

**Results:**

Each 1% increase in lymphocyte (OR 1.01, 95% CI 1.01–1.02, *p* < 0.001) and eosinophil percentage (OR 1.05, 95% CI 1.01–1.11, *p* = 0.01) were independently associated with pneumonitis, while neutrophil percentage was inversely associated (OR 0.99, 95% CI 0.98–0.99, p = 0.01) with pneumonitis. In multivariable analysis, lymphocyte percentage (OR 1.02, 95% CI 1.009–1.04, *p* = 0.002) and eosinophil percentage (OR 1.10, 95% CI 1.01–1.23, *p* = 0.05) were both associated with ICIP. The AUROC for BAL lymphocytes to diagnose ICIP was 0.62 (95% CI 0.57–0.67, optimal cutoff 15.5%, sensitivity 69%, and specificity 52%) and the AUROC for eosinophils was 0.61 (95% CI 0.56–0.66, optimal cutoff 1%, sensitivity 58%, and specificity 62%). In patients with pneumonitis, lymphocyte percentage (HR 0.99, 95% CI 0.97–1.00, *p* = 0.02), neutrophil percentage (HR 1.01, 95% CI 1.00–1.02, *p* = 0.05), and eosinophil percentage (HR 0.93, 95% CI 0.86–0.99, *p* = 0.03) were associated with 1-year survival.

**Conclusion:**

BAL lymphocytosis and eosinophilia are associated with ICIP, but their ability to discriminate ICIP from other conditions is modest. BAL immune cell percentages may have prognostic value for 1-year survival, but this likely reflects the morbidity of other pulmonary diseases that require BAL for evaluation.

## Introduction

Immune checkpoint inhibitors (ICIs) have dramatically altered the landscape of cancer therapies, and they are integral to therapeutic regimens in many solid organ and hematologic malignancies (1). ICIs have improved clinical outcomes and survival in many cancers, but they are also frequently associated with immune-mediated adverse events (iRAEs), which can result in significant harm (2). ICI pneumonitis (ICIP) is of particular concern, since it is the leading cause of mortality related to ICI therapies (3, 4).

A prompt diagnosis of ICIP is essential to limit progression to severe pneumonitis and respiratory failure, particularly since high-grade (3+) pneumonitis often precludes further ICI therapies (5), indirectly increasing the risk of mortality attributable to pneumonitis (6). However, the diagnosis of ICIP is typically based on clinical features, including symptoms, timing of onset, and radiological findings from high-resolution computed tomography (HRCT), such as consolidation and ground-glass opacities (7). However, the clinical presentation of ICIP can mimic other conditions, such as infectious pneumonia or cancer progression. Distinguishing ICIP from other conditions remains a major diagnostic challenge, and biomarkers to diagnose ICIP are lacking.

Bronchoalveolar lavage (BAL) is often performed to evaluate for opportunistic infection, but it is not clear whether BAL cell percentage can also help diagnose ICIP. BAL lymphocytosis is generally not useful in the evaluation of sporadic interstitial lung diseases (8), but small studies have shown that ICIP is associated with BAL lymphocytosis when compared to ICI-treated patients without ICIP (9). We sought to evaluate whether BAL cell percentages, including lymphocytes, were accurate diagnostic markers of ICIP in a cohort of patients with cancers treated with ICIs.

## Methods

### Subjects

We conducted a retrospective review of 476 consecutive patients with both solid organ tumors and hematologic malignancies who were admitted from 2016 to 2023 at The University of Texas MD Anderson Cancer Center with symptoms of acute respiratory symptoms with bilateral infiltrates on imaging, with signs and symptoms of fever, cough, and acute hypoxia. All the subjects had received treatment with anti-programmed cell death protein (PD-1) antibodies either as monotherapy or in combination with an anti-cytotoxic T-lymphocyte-associated protein 4 (CTLA-4) for the treatment of solid organ and hematologic cancer. All patients underwent a bronchoscopy with BAL after ICI initiation. For patients who underwent more than one BAL, data from the first BAL were used. This study was approved by the Institutional Review Board (2022–1083).

### Definitions

We classified cases as ICIP, infectious pneumonia, or other non-infectious etiologies (e.g., cancer progression). ICIP was defined in cases with (1) congruent symptoms (e.g., cough and shortness of breath) and imaging (e.g., patchy ground-glass opacities) and (2) a distinct response to corticosteroids not attributable to antibiotics. Infectious pneumonia was defined based upon (1) congruent symptoms (e.g., cough and shortness of breath) and imaging (e.g., lobar consolidation with air bronchograms) and (2) a clear response to antibiotic treatment not attributable to corticosteroids or isolation of a known pneumonia-causing organism. Other non-infectious causes were individually adjudicated based on the electronic health record. Two independent reviewers who were blinded to cell percentages evaluated all cases based on the preceding criteria. Discrepant cases were reviewed by a third physician who was blinded to the BAL cell percentage and initial diagnosis by the first two readers. Pneumonitis was graded according to the Common Terminology for Cancer Adverse Events 5.0 guidelines (10).

### Data collection

We collected clinical, imaging, and microbiologic data from the electronic medical record. We obtained the types of immunotherapies used, volume instilled and returned at BAL cell percentage with differential, and all microbiologic data, including gram stain, bacterial and fungal cultures, and respiratory virus infections.

### Statistical methods

Logistic regression models were fit to measure the association of BAL cell lines and the diagnosis of ICIP versus other etiologies, both infectious and non-infectious. Effect measure modification analyses were conducted to assess the impact of various modifiers on the relationship between BAL cell lines and pneumonitis. Additionally, multinomial logistic regression was used to evaluate whether pneumonitis exhibited different performance compared to infectious and non-infectious etiologies. The area under the receiver-operator curve (AUROC) was calculated to assess discrimination. For the best-performing models, we calculated the optimal cutoff point, sensitivity, specificity, and area under the curve. We also fit Cox proportional hazard models to measure the association between BAL cell lines and overall survival in the first year after BAL, after adjusting for age and cancer diagnosis. Principal statistical analyses were performed by FS and AS. All authors had access to the complete dataset. All analyses were performed using R, version 4.3.2.

## Results

### Characteristics of the study cohort

Table 1 presents the demographic and clinical characteristics of 476 patients enrolled in the study. The median age was approximately 66 years, and most patients were white (81%) and male (62%). A significant proportion were on corticosteroids (45%) and non-steroidal immunosuppression (27%), such as infliximab or tocilizumab, at the time of BAL. A total 73% of patients were treated with ICIs for solid organ tumors, and the remainder for hematologic malignancies (Table 1). Approximately 6% had evidence of autoimmune diseases at the time of ICI, including pituitary disorders (adrenal insufficiency, hypophysitis, and hypoparathyroidism; *n* = 5), rheumatoid arthritis (*n* = 5), Sjogren disease (*n* = 5), inflammatory bowel disease (*n* = 3), and systemic lupus erythematosus (*n* = 2).

**Table 1:** Patient characteristics and the type of primary malignancy.

Characteristics	*N* = 476 (%)
Age (median [IQR])	66.19 [57.11, 72.15]
Sex, Female	180 (38)
Race	
White or Caucasian	387 (81)
Asian	35 (7)
Black or African American	27 (6)
Other	25 (5)
Declined to answer	1 (0.2)
American Indian or Alaska Native	1 (0.2)
Selected comorbidities	
COPD	111 (23)
ILD	19 (4)
Autoimmune diseases	30 (6)
Primary malignancy	
Solid organ	314 (66)
Non-small cell lung cancer (adenocarcinoma, squamous cell carcinoma)	116 (37)
Genitourinary cancer (renal cell carcinoma, urothelial cancer, prostate cancer)	72 (23)
Gastrointestinal cancer (pancreatic cancer, hepatocellular cancer, cholangiocarcinoma, colon cancer)	30 (10)
Head and neck cancer	20 (6)
Breast cancer	15 (5)
Thyroid cancer	14 (4)
Esophageal cancer	8 (3)
Small cell lung cancer	7 (2)
Gynecological cancer (ovarian, endometrial, and cervical cancer)	7 (2)
Other cancers	36 (12)
Hematologic	162 (34)
Acute myelogenous leukemia	54 (33)
Lymphoma (hodgkin lymphoma, diffuse large b cell lymphoma)	33 (20)
Melanoma	32 (20)
Myelodysplastic syndrome	26 (16)
Chronic myelogenous leukemia	15 (9)
Multiple myeloma	2 (1)

COPD, chronic obstructive pulmonary disease; ILD, interstitial lung disease; BAL, bronchoalveolar lavage; IQR, interquartile range.

### BAL characteristics

The median time from symptom onset to bronchoscopy was 8 days (interquartile range [IQR] 3–20 days). The median volume of BAL fluid instilled was 100 milliliters, while the median volume returned was 40 milliliters. The median percentage of BAL lymphocytes was 19% [IQR 9–37%], and the median percentage of BAL neutrophils was 8% [3.00, 27.00]. A total of 88% of patients had no evidence of BAL eosinophilia. A total of 8% of samples tested positive for a viral panel, 17% for a bacterial culture, and 6% for a fungal culture. Positive results for acid-fast organisms were found in approximately 3% of samples and included: *Mycobacterium abscessus*, *Mycobacterium avium*, *Mycobacterium gordonae*, and *Mycobacterium intracellulare*. BAL immune cell percentages were not significantly correlated with peripheral blood cell percentages, as shown in Supplementary Table 1.

### ICIP characteristics

**Figure 1:**
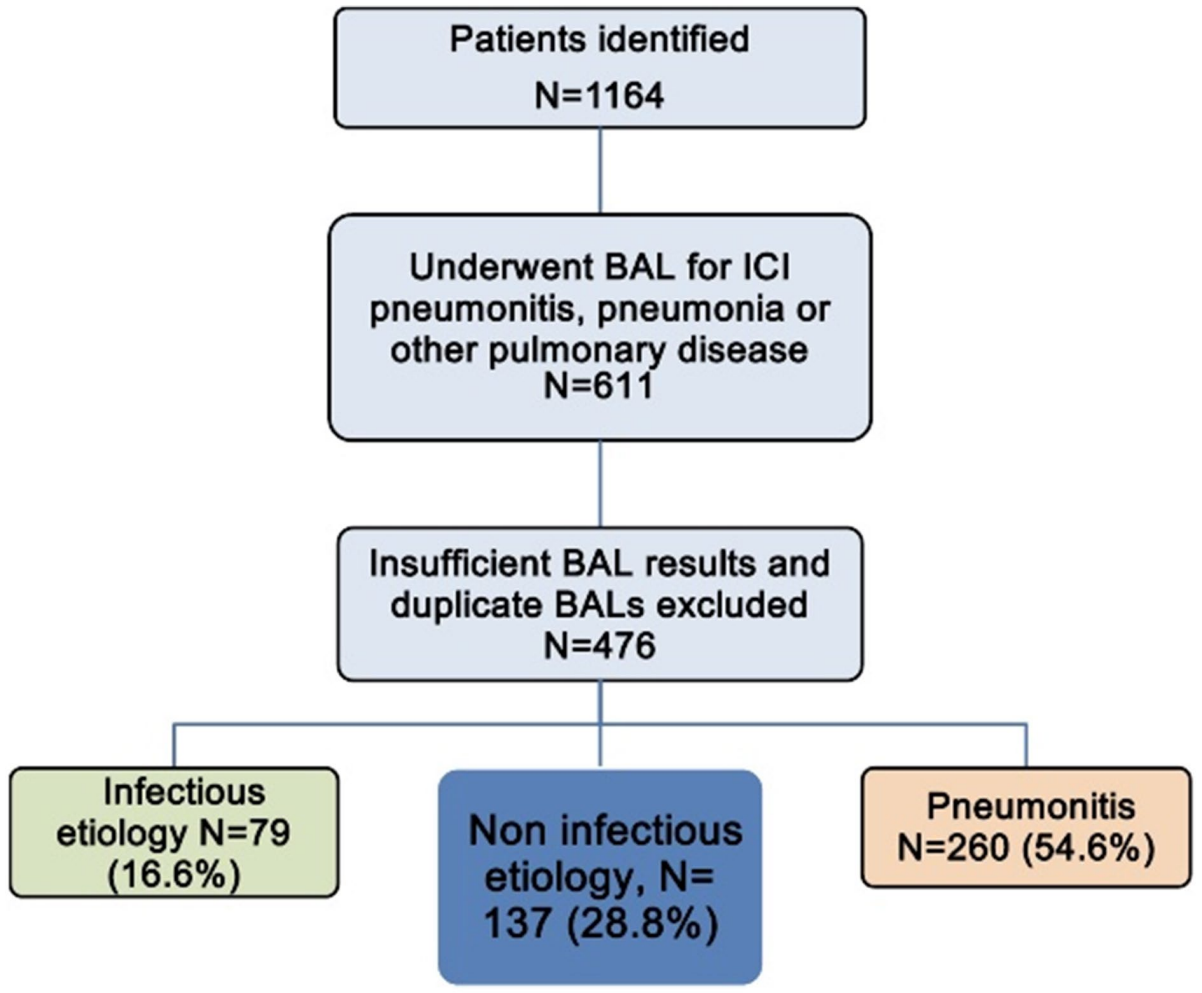
Determination of cohort for pneumonitis, infectious pneumonia, and other non-infectious etiology. BAL, bronchoalveolar lavage; ICI, immune checkpoint inhibitor.

After adjudication, approximately 55% of cases were diagnosed as ICIP, 17% as infectious pneumonia, and 29% as non-infectious etiologies (primarily cancer progression) (Figure 1). In cases of ICIP, 27% of cases had an initial grade of 2, 72% had an initial grade of 3, and 1% with an initial grade of 4. 15% had a maximum grade of 2, 75% had a maximum grade of 3, 9% had a maximum grade of 4, and 1 patient died (grade 5) (Table 2).

**Table 2:** Characteristics from bronchoalveolar lavage (BAL) and grade for verified immune checkpoint inhibitor pneumonitis (ICIP).

BAL characteristics, *n* (median, *n* = 476), IQR (median [IQR])	
Time from symptom onset to bronchoscopy (days)	8.0 [3.0, 20.0]
Volume instilled (ml)	100.0 [80.0, 120.0]
Volume returned (ml)	40.0 [35.0, 50.0]
Cell percentage percentages (median [IQR])	
Lymphocyte %	19.0 [8.7, 37.0]
Neutrophil %	8.0 [3.0, 27.0]
Eosinophil %	0 [0.0, 2.0]
Histiocyte %	45.0 [26.0, 65.0]
Other cell %	3.0 [1.0, 9.0]
Positive microbiologic data, *N* (%)	
Viral panel	40 (8)
Bacterial culture	83 (17)
Fungal culture	30 (6)
Acid-fast stain^	13 (3)
Grading of pneumonitis*	
Initial grade	
2	64 (26)
3	173 (72)
4	2 (1)
Maximum grade	
2	36 (15)
3	180 (75)
4	22 (9)
5	1 (4)

BAL, bronchoalveolar lavage; IQR, interquartile range. ^^^Includes *Mycobacterium abscessus*, *Mycobacterium avium*, *Mycobacterium gordonae*, *Mycobacterium intracellulare*. *Grading of pneumonitis as outlined by the Common Terminology Criteria for Adverse Events v5.0.

### BAL cell percentages in patients with ICIP compared to all others

Table 3 summarizes the association between BAL immune cell percentage and pneumonitis versus all competing etiologies. Each 1% increase in lymphocyte percentage was significantly associated with a diagnosis of ICIP compared to other conditions (OR 1.01, 95% CI 1.009–1.02, *p* < 0.001). Similarly, a 1% increase in eosinophils was associated with a diagnosis of ICIP (OR 1.05, 95% CI 1.01–1.11, *p* = 0.01). Conversely, each 1% increase in neutrophils was associated with a decreased likelihood of ICIP (OR 0.99, 95% CI 0.98–0.99, *p* = 0.01). No significant association was found for histiocytes (OR 0.99, 95% CI 0.99–1.00, *p* = 0.66). In multivariate analyses, each 1% increase in lymphocytes was associated with a diagnosis of ICIP (OR 1.02, 95% CI 1.009–1.04, *p* = 0.002), and each 1% increase in eosinophils was also associated with a diagnosis of ICIP (OR 1.10, 95% CI 1.01–1.23, *p* = 0.05).

**Table 3:** Binary logistic regression models for discrimination of immune checkpoint inhibitor pneumonitis (ICIP) versus all other etiologies by cell line percentage in bronchoalveolar lavage.

Variables	Univariate OR (95% CI)	*p*-value	Multivariate OR (95% CI)	*p*-value
Lymphocytes	1.01 (1.01–1.02)	<0.001	1.01 (1.01–1.02)	<0.001
Neutrophils	0.99 (0.98–0.99)	0.01	–	–
Eosinophils	1.05 (1.01–1.11)	0.01	1.04 (1.00–1.1)	0.04
Histiocytes	0.99 (0.99–1)	0.66	–	–

OR, odds ratio.

The association of absolute cell percentages with ICIP is included in Supplementary Table 2, which presents binary logistic regression models for discrimination of ICIP versus all other etiologies by absolute cell percentage; however, the performance of absolute cell percentages was inferior to that of cell percentage, perhaps owing to variability in dilution.

### BAL cell percentages in ICIP compared to infectious or non-infectious etiologies

Table 4 summarizes the association between BAL immune cell percentages in pneumonitis compared to non-infectious and infectious conditions. Each 1% increase in lymphocyte percentage was associated with an increased likelihood of pneumonitis compared to infectious conditions (OR 1.03, 95% CI 1.01–1.04, *p* < 0.001), but not compared to non-infectious conditions (OR 1.01, 95% CI 0.99–1.02, *p* = 0.236). In other words, the utility of BAL lymphocytosis was highest when distinguishing ICIP from infectious pneumonia. Similarly, a 1% increase in eosinophils was associated with a diagnosis of ICIP compared to infectious conditions (OR 1.13, 95% CI 1.02–1.25, *p* = 0.018), but not significantly compared to non-infectious conditions (OR 1.09, 95% CI 0.99–1.21, *p* = 0.075). Conversely, each 1% increase in neutrophils was associated with a decreased likelihood of ICIP when compared to infectious conditions (OR 0.99, 95% CI 0.98–1.00, *p* = 0.003), but not non-infectious conditions (OR 1.01, 95% CI 0.99–1.02, *p* = 0.354). Histiocytes were not significantly associated with ICIP, infections, or non-infectious etiologies. In summary, the utility of lymphocyte, eosinophil, and neutrophil percentages was highest when distinguishing ICIP from infectious pneumonia.

**Table 4:** Multinomial logistic regression models for discrimination of immune checkpoint inhibitor pneumonitis (ICIP) versus infectious or non-infectious etiologies by cell line percentage in bronchoalveolar lavage.

Variables	Comparison	1/OR (95% CI)	*p*-value
Lymphocytes	Pneumonitis vs. Non-infectious	1.01 (0.99–1.02)	0.236
	Pneumonitis vs. Infectious	1.03 (1.01–1.04)	<0.001
Neutrophils	Pneumonitis vs. Non-infectious	1.01 (0.99–1.02)	0.354
	Pneumonitis vs. Infectious	0.99 (0.98–1.00)	0.003
Eosinophils	Pneumonitis vs. Non-infectious	1.09 (0.99–1.21)	0.075
	Pneumonitis vs. Infectious	1.13 (1.02–1.25)	0.018
Histiocytes	Pneumonitis vs. Non-infectious	0.99 (0.98–1.00)	0.046
	Pneumonitis vs. Infectious	1.00 (0.99–1.01)	0.559

The association of absolute cell percentages with ICIP in multinomial logistic regression models to discriminate ICIP versus infectious or non-infectious etiologies is included in Supplementary Table 3, but the performance is inferior than with cell percentage.

### Performance of BAL cell percentages after adjusting for steroid use and type of malignancy

We constructed several logistic regression models to evaluate potential effect-measure modifiers on the association between BAL cell percentages and ICIP. In a model evaluating the impact of steroid use on the association of BAL lymphocytosis with ICIP, steroid use was associated with ICIP (OR 2.2, 95% CI 1.2–4.0, *p* = 0.01), but lymphocyte percentage remained significantly associated with ICIP (OR 1.03, 95% CI 1.01–1.04, *p* < 0.001), with no evidence of effect-measure modification (*p* = 0.363 for interaction).

In a model examining type of malignancy (solid organ vs. hematologic) and BAL lymphocytosis, hematologic malignancy was associated with a lower risk of ICIP (OR 0.3, 95% CI 0.1–0.5, p < 0.001), while lymphocyte percentage remained significantly associated with ICIP (OR 1.02, 95% CI 1.01–1.03, *p* = 0.003). There was no evidence of effect-measure modification (*p* = 0.8 for interaction).

We constructed two similar models to assess the association of eosinophil percentage with ICIP while adjusting for steroid use and type of malignancy. In the model examining steroid use, neither steroid use (OR 1.4, 95% CI 0.9–2.1, *p* = 0.12) nor eosinophil percentage (OR 1.04, 95% CI 1.00–1.10, *p* = 0.07) was significantly associated with ICIP, but there was evidence of effect-measure modification (*p* = 0.048 for interaction). Therefore, we performed stratified analyses by steroid use at the time of BAL. In the steroid-treated group, the eosinophil percentage was associated with ICIP (OR 1.23, 95% CI 1.07–1.47, *p* = 0.009). In the group that was not treated with steroids, the association was weaker and not statistically significant (OR 1.04, 95% CI 1.00–1.10, p = 0.07). Eosinophil percentages also differed between the groups, with steroid-treated patients showing a median of 0 and a mean of 1.5%, while untreated patients had a median of 1 and a mean of 2.9%.

In models examining malignancy type and eosinophil percentage, hematologic malignancy was associated with a lower risk of ICIP (OR 0.2, 95% CI 0.1–0.4, *p* < 0.001), but eosinophil percentage was not (OR 1.02, 95% CI 0.98–1.08, *p* = 0.28), with no evidence of effect-measure modification (*p* = 0.17 for interaction).

### Diagnostic performance of BAL cell percentages for pneumonitis

**Figure 2:**
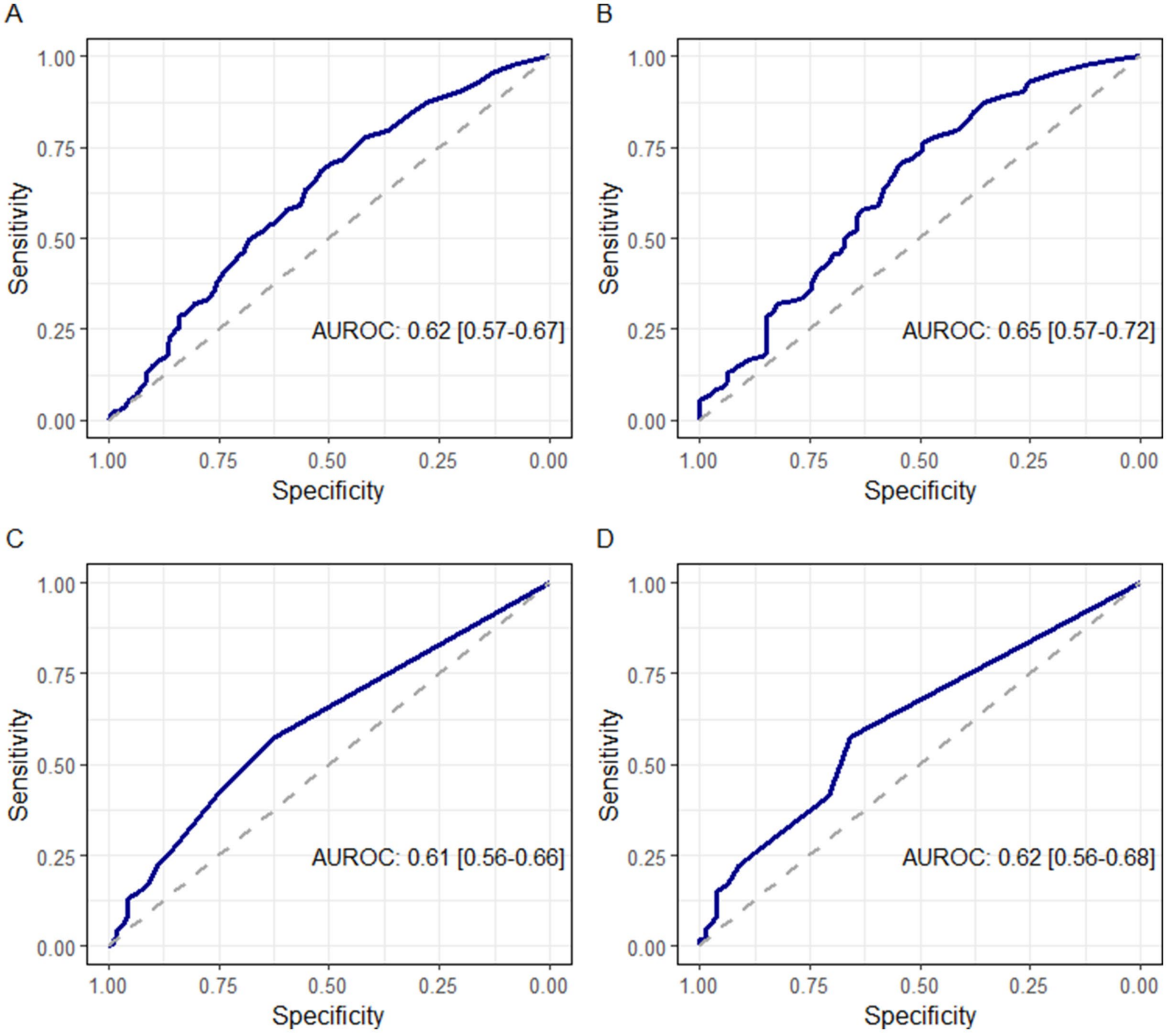
Diagnostic performance of bronchoalveolar lavage (BAL) cell lines for all patients. Panel **A** shows the receiver operating characteristics (ROC) curve describes the diagnostic performance of BAL lymphocyte percentage for pneumonitis versus all other etiologies. Panel **B** shows the ROC curve describing the diagnostic performance of BAL lymphocyte percentage for pneumonitis versus infectious etiologies. Panel **C** shows the ROC curve describing the diagnostic performance of BAL eosinophil percentage for pneumonitis versus all other etiologies. Panel **D** shows the ROC curve describing the diagnostic performance of BAL eosinophil percentage for pneumonitis versus infectious etiologies.

Figure 2 summarizes the diagnostic performance of lymphocyte and eosinophil percentage on BAL for all patients, while Supplementary Figure 1 summarizes the diagnostic performance of lymphocyte percentage and eosinophil percentage on BAL, stratified by type of malignancy (solid versus hematological). The AUROC of BAL lymphocyte percentage to distinguish pneumonitis from all other causes yielded an AUROC of 0.62 (95% CI 0.57–0.67). The optimal cutoff was 15.5%, with a sensitivity of 69% and a specificity of 52%. When differentiating ICIP from infectious etiologies, the AUC for BAL lymphocyte percentage was 0.65 (95% CI 0.57–0.72), with an optimal cutoff of 11.5%, a sensitivity of 76%, and a specificity of 49%. For BAL eosinophil percentage, the AUC for diagnosing ICIP versus all other causes was 0.61 (95% CI 0.56–0.66), with an optimal cutoff of 1%, a sensitivity of 58%, and a specificity of 62%. Similarly, for distinguishing ICIP from infectious etiologies, the AUC was 0.61 (95% CI 0.56–0.66), with the same optimal cutoff of 1%, sensitivity of 58%, and specificity of 62%.

Supplementary Table 4 summarizes the sensitivity analysis to evaluate the diagnostic performance of BAL lymphocyte and eosinophil percentages for distinguishing pneumonitis from other etiologies, after excluding participants with autoimmune diseases and those receiving systemic corticosteroids at the time of bronchoscopy. When excluding individuals with autoimmune conditions, the AUROCs for lymphocytes were 0.63 (95% CI: 0.58–0.64) for pneumonitis vs. all other causes and 0.65 (0.56–0.74) for pneumonitis vs. pneumonia. Eosinophils yielded AUROCs of 0.62 (0.57–0.68) and 0.65 (0.58–0.72), respectively. After excluding patients receiving steroids, the AUROC for lymphocytes increased to 0.66 (0.59–0.72) and 0.72 (0.63–0.82), and for eosinophils to 0.63 (0.56–0.69) and 0.63 (0.54–0.72), for the same comparisons. These results suggest that the diagnostic utility of BAL differential cell counts, particularly for lymphocytes, may be enhanced in the absence of immunosuppressive therapies.

### Association of BAL cell percentages with 1-year survival

Because pneumonitis has been associated with mortality (6, 11), we measured the association of BAL cell percentages with 1-year survival and stratified by pneumonitis versus other conditions. In patients with pneumonitis, lymphocyte percentage (HR 0.99, 95% CI 0.97–1.00, *p* = 0.02), neutrophil percentage (HR 1.01, 95% CI 1.00–1.02, *p* = 0.05), and eosinophil percentage (HR 0.93, 95% CI 0.86–0.99, *p* = 0.03) were associated with 1-year survival, while histiocytes were not (HR 1.00, 95% CI 0.99–1.01, *p* = 0.91). In patients without pneumonitis, none of the cell percentages were significantly associated with 1-year survival: lymphocyte percentage (HR 1.00, 95% CI 0.99–1.01, *p* = 0.79), neutrophil percentage (HR 1.00, 95% CI 0.99–1.00, *p* = 0.29), eosinophil percentage (HR 0.94, 95% CI 0.86–1.02, *p* = 0.14), or histiocyte percentage (HR 1.01, 95% CI 1.00–1.02, *p* = 0.13). Among patients with ICIP who were treated with steroids, lymphocyte percentage (HR 0.98, 95% CI 0.97–1.00, *p* = 0.06) and neutrophil percentage (HR 1.01, 95% CI 1.00–1.02, *p* = 0.08) were not significantly associated with 1-year survival, but the direction of effect was similar to all patients with ICIP. On the other hand, eosinophil percentage (HR 0.98, 95% CI 0.89–1.07, *p* = 0.60) and histiocyte percentage (HR 1.00, 95% CI 0.98–1.01, *p* = 0.52) had no measurable association with 1-year survival. In pneumonitis patients not treated with steroids, the eosinophil percentage was not significantly associated with 1-year survival, but the direction of effect was similar to all patients with ICIP (HR 0.89, 95% CI 0.79–1.00, *p* = 0.05). None of the other cell percentages were associated with 1-year survival: lymphocyte percentage (HR 0.99, 95% CI 0.97–1.01, *p* = 0.33), neutrophil percentage (HR 1.01, 95% CI 0.99–1.03, *p* = 0.49), and histiocyte percentage (HR 1.00, 95% CI 0.99–1.02, *p* = 0.93).

## Discussion

In this study, we showed that BAL lymphocyte and eosinophil percentages are associated with a diagnosis of ICIP, and conversely, that BAL neutrophilia is associated with a diagnosis other than ICIP. However, the ability of these markers to diagnose ICIP is limited. The association of BAL lymphocytosis and eosinophilia with pneumonitis was similar in solid organ and hematologic malignancies, and it was most useful in distinguishing ICIP from pneumonia. However, the clinical utility of BAL cell percentages on their own is likely to be limited given the subpar sensitivity and specificity of these routine tests, indicating the need for enhanced biomarkers. Finally, we observed novel associations between BAL immune cell percentages and survival following ICIP.

BAL cell percentages are routine labs that are often useful in certain diagnostic scenarios, such as the identification of eosinophilic lung diseases (12). Pulmonary infections are often associated with BAL neutrophilia (13) but BAL neutrophil percentage is rarely used to make clinical decisions. BAL lymphocytosis only has a limited value in distinguishing between subtypes of sporadic interstitial lung diseases (14). However, others have shown the association of BAL lymphocytosis with a diagnosis of ICIP. In a study of 18 ICI-treated patients, of whom 12 developed ICI pneumonitis, the ratio of CD4 + to CD8 + T-lymphocytes was not different in those with and without ICIP, but ICIP was associated with an increase in BAL absolute lymphocyte percentages (9). A similar association was seen in a study of 7 patients with acute myeloid leukemia who were treated with ICIs and developed either pneumonia or ICIP; however, an expansion of Th17.1 cells was seen in all ICI-treated patients but did not distinguish ICIP from pneumonia (15). On the other hand, in a study of 11 patients with ICIP compared to 6 patients who were not treated with ICI, Th17.1 cells accounted for 13% of all immune cells in BAL fluid among those with ICIP, compared to only 3% in controls (16). Our findings support the association of lymphocytosis with ICIP in a much larger cohort and with the use of conventional laboratory measurements of BAL lymphocyte percentage. However, similar to idiopathic interstitial lung diseases, the enthusiasm for the use of lymphocytosis as a biomarker on its own is tempered by its limited sensitivity and specificity.

While most studies focus on the association of T-lymphocytes with irAEs, including ICIP, comparatively few have explored the role of eosinophils in irAEs. In a study of 13 patients treated with ICI, of whom 7 developed ICIP, BAL fluid in patients with ICIP showed evidence of a type 1 skew (17). No studies to date suggest a type 2 skew in BAL inflammation, though a recent study suggested that patients with ICIP had evidence of a Th2 predominance in peripheral blood lymphocytes (18). Peripheral eosinophilia is evident in a significant minority of patients undergoing ICI treatment, but little data exist about BAL eosinophilia. Though we found that BAL eosinophil percentage was associated with ICIP, we suggest that BAL eosinophil percentage is a suboptimal diagnostic biomarker for ICIP for two reasons. First, the optimal cutoff in our study was 1%, and few people had any evidence of eosinophilia. Therefore, while our data suggest that any degree of BAL eosinophilia is associated with a diagnosis of ICIP, most patients with ICIP will not have evidence of eosinophilia. Second, BAL eosinophil percentage was less powerful as a biomarker to distinguish ICIP from infectious or non-infectious etiologies, likely owing to the overall rarity of BAL eosinophilia in the cohort. Third, the utility of BAL eosinophil percentage is affected by the presence of steroids at the time of BAL, though our data suggest that BAL eosinophilia that is evident in patients treated with steroids is more strongly associated with ICIP. One possibility is that eosinophils undergo more rapid apoptosis with the use of steroids compared to lymphocytes, and therefore, the persistence of eosinophilia despite steroids is a marker of ICIP activity, but this speculation would need to be confirmed with mechanistic studies (19). In short, we do not recommend the use of BAL eosinophil percentages as a reliable diagnostic biomarker for pneumonitis.

Nevertheless, we observed provocative associations between BAL immune cell percentages at the time of BAL and subsequent survival. We found no association of BAL immune cell percentages with survival in patients without ICIP, but in those with ICIP, BAL lymphocytosis and eosinophilia were associated with improved survival, while neutrophilia was associated with poorer survival. These effects diminished when stratifying by the presence of steroids at the time of BAL, though this was limited by the smaller sample of each subgroup. Others have found an association between peripheral eosinophilia and survival. In a study of 430 patients with lung cancer receiving ICI, of whom 16% developed ICIP, increasing peripheral eosinophil percentages were associated with pneumonitis incidence and improved overall survival (20). Similarly, in a study of 300 patients with advanced non-small cell lung cancer, of whom 18% developed pneumonitis, pre-ICI peripheral eosinophilia predicted pneumonitis and was associated with improved progression-free survival (21). Peripheral lymphocytosis, often measured in the context of the neutrophil-to-lymphocyte ratio, is associated with a higher risk of irAEs (22), including pneumonitis, and improved survival (23). Peripheral and BAL immune cell percentages may correlate to some degree in some settings (24), but we found no significant correlations in our study, and therefore, studies comparing peripheral blood immune cells and BAL immune cells should not necessarily be considered equivalent. To our knowledge, no study has examined the association of BAL immune cell percentages with survival. These findings must also be interpreted in the context of which patients undergo BAL and the timing of corticosteroid therapy. Corticosteroids promote apoptosis in eosinophils but inhibit apoptosis in neutrophils (25). Nearly half of the cohort was on corticosteroids at the time of BAL, perhaps because our cohort consisted of mostly grade 3 pneumonitis, and therefore, we cannot fully rule out the possibility of confounding despite our strategy for stratification. More studies are needed to fully understand these findings.

While our studies show that BAL cell percentages may have limited value to differentiate pneumonitis from other conditions, there may be value in using this information to augment additional tools. In a study of 126 patients with acute myeloid leukemia treated with ICIs, in which pneumonia was far more common than ICIP, the use of a radiomic strategy called “habitat imaging” was able to distinguish pneumonia from ICIP with an accuracy of 79% (26). However, integrating information from blood markers improved the accuracy to 81%, increasing both sensitivity and specificity. Although BAL immune cell percentages, including lymphocyte percentages, are routinely assessed and widely accessible, their diagnostic utility for ICIP remains limited when used as a standalone measure. However, BAL immune cell percentages may serve as a complementary tool to enhance the diagnostic accuracy of ICIP when integrated with more robust diagnostic modalities. In other words, BAL lymphocytosis, while non-specific on its own, may augment the diagnostic performance of other tools or markers used to identify ICIP. Future studies are warranted to explore whether specific immune cell subtypes or cytokine profiles within BAL fluid can provide additional diagnostic specificity for ICIP, distinguishing it from infectious etiologies or other forms of interstitial lung disease.

Some limitations exist. First, our retrospective design may introduce selection bias, particularly since only patients who underwent bronchoscopy could be included. This restricts the patients who developed pneumonitis to a narrow range of severities—those that were sick enough to warrant BAL, but not too sick to deteriorate after BAL, and this narrow range is reflected in the initial grade of pneumonitis severity (mostly grade 2 or 3). Second, while the diagnostic criteria used for pneumonitis were based on established clinical and imaging features, the lack of a definitive histologic confirmation for most cases may have led to misclassification in a minority of cases. Third, given the retrospective nature of the study, we could not account for confounding by indication (for example, the association between steroid use and pneumonitis). Additionally, we could not account for all potential confounders, such as specific immunosuppressive therapies that were included as part of the cancer regimens, because this is a cohort of patients with a diverse array of cancers. However, our study has several strengths. Our cohort was a comprehensive assessment of all ICI-treated patients who underwent BAL at our institution and is the largest cohort to examine the association of BAL lymphocyte percentage with ICIP. Adjudication of pneumonia and ICIP was performed systematically by experienced readers. We compared the performance of BAL immune cell differentials in several relevant scenarios, such as in solid tumor versus hematologic malignancies, and among patients with or without steroid exposure at the time of BAL. In our cohort, 4% of patients had ILD, which included both clinically significant ILD and ILAs noted in the medical record. We did not systematically differentiate between ILAs and fibrotic ILD, and radiation fibrosis was only included if explicitly documented. This lack of granularity is a limitation, particularly given the known association between ILD and increased risk of ICIP (9). Due to the small sample size, we could not perform subgroup analyses, and future studies should apply standardized criteria to better characterize ILD subtypes in this context.

## Conclusion

In conclusion, we show that BAL lymphocytosis and eosinophilia are associated with a diagnosis of ICIP but have limited diagnostic utility owing to low sensitivity and specificity. Future research should focus on combining BAL immune cell subsets with other potential biomarkers, including imaging, or considering specific diagnostic biomarkers that can be obtained from BAL. Early and accurate differentiation of pneumonitis from other causes of respiratory distress remains a critical challenge, and further studies are needed to refine diagnostic strategies for patients on ICI therapy.

## Data Availability

The datasets presented in this article are not readily available because they contain protected health information and are subject to institutional privacy policies and IRB restrictions (IRB protocol # 2022-1083). De-identified data may be made available upon reasonable request, pending appropriate data use agreements and institutional approvals. Requests to access the datasets should be directed to Asheshadri@mdanderson.org.
