# Correction: Bronchoalveolar lavage cell percentages as diagnostic markers of immune checkpoint inhibitor pneumonitis

**DOI:** 10.3389/fmed.2025.1646108

**Published:** 2025-06-26

**Authors:** Mahnoor Mir, Felipe Soto, Pedro Antonio Amezcua Gomez, Rodrigo Del Rio Arroyo, Adarsh Suresh, Amber Su, Qiong Gan, John Stewart, Roberto Adachi, Diwakar D. Balachandran, Lara Bashoura, Roberto F. Casal, Burton F. Dickey, George A. Eapen, Scott E. Evans, Horiana Grosu, Carlos A. Jimenez, Julie Lin, David E. Ost, Bruce F. Sabath, Vickie R. Shannon, Aung Naing, Jianjun Gao, Jia Wu, Karthik Suresh, Saadia A. Faiz, Mehmet Altan, Ajay Sheshadri

**Affiliations:** ^1^Division of Critical Care, Pulmonary, and Sleep Medicine, McGovern Medical School at UTHealth Houston, Houston, TX, United States; ^2^School of Medicine, Tecnológico de Monterrey, Monterrey, Mexico; ^3^Texas A&M School of Medicine, Houston, TX, United States; ^4^Department of Pulmonary Medicine, The University of Texas MD Anderson Cancer Center, Houston, TX, United States; ^5^Department of Pathology, The University of Texas MD Anderson Cancer Center, Houston, TX, United States; ^6^Department of Investigational Cancer Therapeutics, The University of Texas MD Anderson Cancer Center, Houston, TX, United States; ^7^Division of Cancer Medicine, Department of Genitourinary Medical Oncology, Houston, TX, United States; ^8^Department of Imaging Physics, The University of Texas MD Anderson Cancer Center, Houston, TX, United States; ^9^Department of Pulmonary and Critical Care Medicine, Johns Hopkins University, Baltimore, MD, United States; ^10^Department of Thoracic Oncology, The University of Texas MD Anderson Cancer Center, Houston, TX, United States

**Keywords:** immune check inhibitor (ICI), BAL (bronchoalveolar lavage), lymphocytosis, pneumonitis, eosinophilia, cell percentage

Author Felipe Soto was erroneously not listed as co-first author in the published article. Author Mehmet Altan was erroneously not listed as co-senior author in the published article. The correct author list reads:

Mahnoor Mir^1^^*^^†^, Felipe Soto^2^^†^, Pedro Antonio Amezcua Gomez^2^, Rodrigo Del Rio Arroyo^2^, Adarsh Suresh^3^, Amber Su^4^, Qiong Gan^5^, John Stewart^5^, Roberto Adachi^4^, Diwakar D. Balachandran^4^, Lara Bashoura^4^, Roberto F. Casal^4^, Burton F. Dickey^4^, George A. Eapen^4^, Scott E. Evans^4^, Horiana Grosu^4^, Carlos A. Jimenez^4^, Julie Lin^4^, David E. Ost^4^, Bruce F. Sabath^4^, Vickie R. Shannon^4^, Aung Naing^6^, Jianjun Gao^7^, Jia Wu^8^, Karthik Suresh^9^, Saadia A. Faiz^4^, Mehmet Altan^10^^‡^ and Ajay Sheshadri^4^^‡^

^†^These authors share first authorship

^‡^These authors share senior authorship

The Author contributions Statement has been corrected to read:

