# Supplementary material for: Bronchoalveolar lavage cell percentages as diagnostic markers of immune checkpoint inhibitor pneumonitis
Source: Front Med (Lausanne). 2025 Jun 2;12:1582714. doi: 10.3389/fmed.2025.1582714 (PMC12171021; doi:10.3389/fmed.2025.1582714)
Supplement: Supplementary file 1 [file Supplementary_file_1.docx]

**Supplemental Figure 1.** Diagnostic Performance of Bronchoalveolar Lavage (BAL) Cell Lines Stratified by Type of Malignancy.


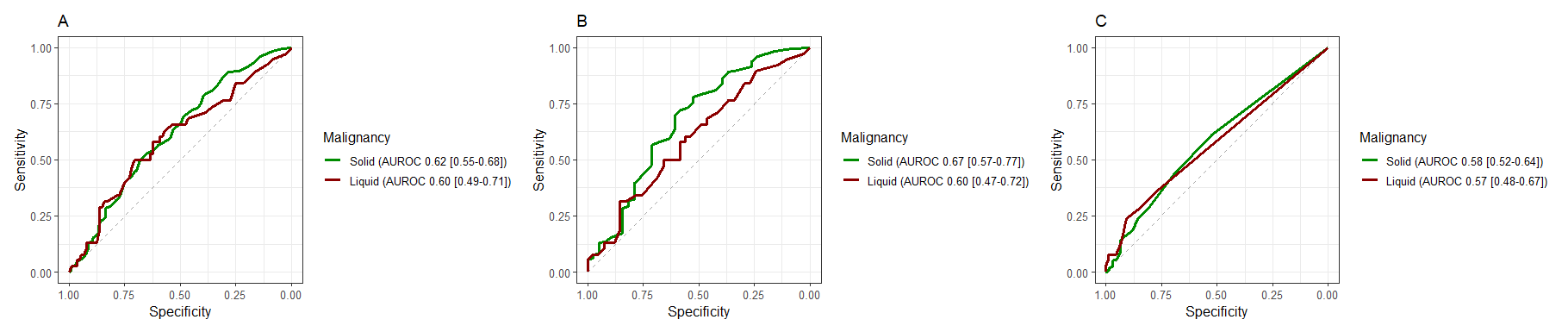


Panel A shows the receiver-operating-characteristics (ROC) curve describing the diagnostic performance of BAL lymphocyte percentage for pneumonitis versus all other etiologies. Panel B shows the ROC curve describing the diagnostic performance of BAL lymphocyte percentage for pneumonitis versus infectious etiologies. Panel C shows the ROC curve describing the diagnostic performance of BAL eosinophils percentage for pneumonitis versus all other etiologies.

**Supplemental Table 1.** Correlation Between Peripheral Complete Blood Count Cell Percentages and Bronchoalveolar Lavage Cell Percentages

| **Variable Pair** | **Correlation (r)** | **p-value** | **95% Confidence Interval** |
| --- | --- | --- | --- |
| Lymphocytes | 0.0307 | 0.5323 | [-0.0656, 0.1265] |
| Eosinophils | -0.0633 | 0.1990 | [-0.1587, 0.0333] |
| Histiocytes | 0.0951 | 0.0525 | [-0.0010, 0.1895] |
| Neutrophils | 0.0784 | 0.1105 | [-0.0179, 0.1732] |

**Supplemental Table 2.** Binary Logistic Regression Models for Discrimination of Immune Checkpoint inhibitor Pneumonitis (ICIP) versus All Other Etiologies by Absolute Cell Line Count in Bronchoalveolar Lavage (BAL)

| **Variables** | **Univariate OR (95% CI)** | **p-value** |
| --- | --- | --- |
| Lymphocytes (K/uL) | 1.002 (0.9995–1.005) | 0.127 |
| Neutrophils (K/uL) | 0.999 (0.9981–0.9998) | 0.027 * |
| Eosinophils (K/uL) | 1.005 (0.9922–1.0185) | 0.487 |
| Histiocytes (K/uL) | 0.999 (0.9974–1.0011) | 0.416 |

**Supplemental Table 3.** Multinomial Logistic Regression Models for Discrimination of Immune Checkpoint inhibitor Pneumonitis (ICIP) versus Infectious or Non-Infectious Etiologies by Absolute Cell Count by Line in Bronchoalveolar Lavage (BAL)

| **Variables** | **1/OR (95% CI)** | **p-value** |
| --- | --- | --- |
| Lymphocytes (K/uL) | 1.002 (0.998–1.01) | 0.493 |
|  | 1.006 (0.996–1.011) | 0.038 |
| Neutrophils (K/uL) | 1.001 (0.998–1.004) | 0.526 |
|  | 1.00 (1.00–1.00) | 0.053 |
| Eosinophils (K/uL) | 1.013 (0.978–1.043) | 0.386 |
|  | 1.016 (0.982–1.044) | 0.267 |
| Histiocytes (K/uL) | 1.002 (0.998–1.006) | 0.312 |
|  | 1.00 (0.999–1.004) | 0.825 |

**Supplemental Table 4.** Sensitivity analysis of BAL Cell lines excluding auto-immune disease and steroids use at the time of bronchoscopy

| **Sensitivity Analysis** | **BAL Marker** | **Comparison** | **AUROC (95% CI)** |
| --- | --- | --- | --- |
| Excluding autoimmune disease | Lymphocytes | Pneumonitis vs All Other Causes | 0.63 (0.58 to 0.64) |
|  | Lymphocytes | Pneumonitis vs Pneumonia | 0.65 (0.56 to 0.74) |
|  | Eosinophils | Pneumonitis vs All Other Causes | 0.62 (0.57 to 0.68) |
|  | Eosinophils | Pneumonitis vs Pneumonia | 0.65 (0.58 to 0.72) |
| Excluding subjects who received steroids | Lymphocytes | Pneumonitis vs All Other Causes | 0.66 (0.59 to 0.72) |
|  | Lymphocytes | Pneumonitis vs Pneumonia | 0.72 (0.63 to 0.82) |
|  | Eosinophils | Pneumonitis vs All Other Causes | 0.63 (0.56 to 0.69) |
|  | Eosinophils | Pneumonitis vs Pneumonia | 0.63 (0.54 to 0.72) |

Area under the receiver operating characteristic curve (AUROC) values for BAL lymphocyte and eosinophil percentages in distinguishing pneumonitis from other etiologies. Analyses were repeated after excluding subjects with a history of autoimmune disease and those receiving systemic corticosteroids at the time of bronchoscopy. Diagnostic performance remained consistent, with improved AUROCs observed particularly for lymphocytes after excluding steroid-treated patients. Values are presented as AUROC (95% confidence interval).
